# Crystal Structures and Piezoelectric Properties of Quenched and Slowly-Cooled BiFeO_3_-BaTiO_3_ Ceramics

**DOI:** 10.3390/ma17184492

**Published:** 2024-09-13

**Authors:** Su Hwan Go, Kang San Kim, Ye Rok Choi, Jeong-Seog Kim, Chae Il Cheon

**Affiliations:** 1 Department of Materials Science & Engineering, Hoseo University, Asan 31499, Republic of Korea; gsh24@naver.com (S.H.G.); nice960203@naver.com (K.S.K.); kimjungs@hoseo.edu (J.-S.K.); 2 Department of Electronic Materials Engineering, Hoseo University, Asan 31499, Republic of Korea; chlwltks49@naver.com

**Keywords:** BiFeO_3_-BaTiO_3_ ceramics, crystal structure, piezoelectric property, quenching

## Abstract

The BiFeO_3_-BaTiO_3_ (BF-BT) ceramics were here prepared through the solid-state reaction of Bi_2_O_3_, Fe_2_O_3_ and nano-sized BT powders. The crystal structures and piezoelectric properties were investigated in both quenched (AQ) and slowly cooled (SC) 0.7BF-0.3BT ceramics. Prior work has shown that rhombohedral and pseudo-cubic phases coexist in 0.7BF-0.3BT ceramics. In this work, the crystal structure of the pseudo-cubic phase was refined as a non-polar orthorhombic Pbnm phase in the SC sample and as a polar orthorhombic Pmc2_1_ phase in the AQ sample. In addition to a sharp dielectric peak at about 620 °C, corresponding to the Curie temperature of the rhombohedral phase, a broad dielectric peak with strong frequency dispersion and a sharp frequency-independent dielectric peak were observed at around 500 °C in the SC and AQ samples, respectively. We determine that the dielectric anomalies around 500 °C were caused by a relaxor phase transition of the non-polar orthorhombic phase in the SC sample and a ferroelectric–paraelectric phase transition of the polar orthorhombic phase in the AQ sample. The AQ sample showed better ferroelectric and piezoelectric properties than the SC sample. The 0.7BF-0.3BT ceramic slowly cooled in a nitrogen atmosphere showed a well-saturated P-E curve and a similar temperature-dependent dielectric constant as the AQ sample. Our results indicate that large concentrations of oxygen vacancies produce a more distorted polar orthorhombic phase and better piezoelectric properties in the AQ sample than in the SC sample.

## 1. Introduction

Piezoelectric materials are widely used in various electronic devices including sensors, actuators, and transducers [1,2,3]. Although lead-based materials such as Pb(Zr,Ti)O_3_ and Pb(Mg,Nb)O_3_-PbTiO_3_ are commonly used in most piezoelectric devices, the presence of Pb in these materials raises significant human health concerns [1,2,3]. As a result, lead-free piezoelectric ceramics such as (K,Na)NbO_3_-LiSbO_3_ and (Bi,Na)TiO_3_-BaTiO_3_ have been developed as alternative materials for the replacement of lead-containing piezoelectric ceramics [2,3,4,5].

Recently, the BiFeO_3_-BaTiO_3_ (BF-BT) ceramic has attracted significant attention as a lead-free material used in high-temperature piezoelectric devices owing to its excellent piezoelectric properties and high Curie temperatures of over 400 °C [5,6,7,8,9]. The crystal structure of the (1 − *x*)BF-*x*BT solid solution has been reported to change from a rhombohedral (R3c) phase at *x* = 0 to a pseudo-cubic around *x* = 0.3 [10,11,12,13,14,15,16,17,18,19,20,21]. In addition, a second phase change from a pseudo-cubic phase to a tetragonal (P4mm) phase occurs at about *x* = 0.9 with increasing BT mole fraction (*x*) [10]. The highest piezoelectric constant (d_33_) has been observed around the morphotropic phase boundary (MPB) between the rhombohedral and pseudo-cubic structures [11,12]. Both a rhombohedral and a pseudo-cubic phase have been reported to coexist at the MPB [7,9,12,13,14,15,16,17,18,19,20,21]. In addition, the pseudo-cubic phase at the MPB has been refined into a cubic structure (Pm-3m) in prior work [7,9,12,13,14,15,16,17,18,19,20,21]. Previous reports have described the pseudo-cubic phase as a relaxor ferroelectric with a broad dielectric peak, which shifts to lower temperatures when the measuring frequency decreases [16,17]. However, some researchers have claimed that a rhombohedral (R3c) phase coexists with a tetragonal phase (P4mm) or a monoclinic (Cm) phase at the MPB [6,22,23]. The crystal structure of the pseudo-cubic phase at the MPB is controversial. Achieving accurate structure refinement for BF-BT ceramics with compositions around the MPB is challenging due to extremely small distortions from the cubic phase. Nevertheless, the dielectric behavior of BF-BT around the phase transition temperature may be helpful for accurately identifying the crystal structure.

The cooling rate has been reported to greatly influence the crystal structure, dielectric behavior and piezoelectric properties of BF-BT ceramics with compositions at the MPB [5,6,16,17,24,25,26,27,28,29,30,31,32]. Quenched BF-BT ceramics showed better saturated ferroelectric polarization–electric field (P-E) hysteresis curves and superior piezoelectric properties compared to slowly cooled samples [5,6,16,17,24,25,26,27,28,29,30,31,32]. The enhanced piezoelectric properties in the quenched BF-BT ceramic, referred to as the “quenching effect”, have been explained through several different mechanisms [6,25,27,32,33,34,35]. These explanations include improved chemical homogeneity to inhibit the formation of a core–shell structure [16,33,34], suppression of the impurity phase and a reduction in the Fe^+2^ ion concentration [6,32], freezing randomly oriented defect dipoles and relaxation of lattice strain [27,35], and crystal structure modification by the diffusion of oxygen vacancies [25].

Recently, a BF-BT ceramic with no impurity phase and less ionic defects was prepared from nano-sized BT powder, and showed a large density and a high piezoelectric constant of over 200 pC/N [36].

In this work, homogeneous 0.7BF-0.3BT ceramics were prepared by the solid-state reaction of Bi_2_O_3_, Fe_2_O_3_ and nano-sized BT powders. Then, we determined the crystal structures for the quenched and slowly cooled samples from Rietveld refinement of X-ray diffraction data. To explore the phase transition behavior, we measured the temperature dependence of the dielectric constant. In addition, the ferroelectric polarization–electric field (P-E) hysteresis curves and piezoelectric properties were measured. We have also studied the effect of the cooling rate on phase evolution and piezoelectric properties, and discussed the origin of the quenching effect.

## 2. Experimental

The 0.7BF-0.3BT ceramics were prepared via a conventional ceramic process with starting materials of Bi_2_O_3_ (Sigma Aldrich, 99.9%, Saint Louis, MO, USA), Fe_2_O_3_ (Sigma Aldrich, 99%) and nano-sized BaTiO_3_ (Sigma Aldrich, <100 nm, 99%). The mixture of raw materials was ball-milled in ethanol with yttria-stabilized zirconia balls for 24 h. After drying the mixed slurry, the powder was calcined in two steps: first at 750 °C for five hours and then at 850 °C for three hours. The calcined powders were ball-milled with 0.2 mol % MnO_2_ (Sigma Aldrich, 99%) for 24 h to improve the insulation resistance. After drying the ball-milled slurry, the powders were compacted in a 12 mm-diameter mold by applying a uniaxial pressure of approximately 90 MPa. The compacted powders were sintered at a temperature of 1020 °C for three hours. The samples were heated to the sintering temperature at a rate of 5 °C/minute and cooled in a furnace. Silver paste was printed on the surface of the samples and heat-treated at 800 °C for 15 min. The SC samples were slowly cooled at rate of 2 °C/minute in the furnace in air after the heat treatment of the silver paste, whereas the AQ samples were removed from the furnace and quenched in air.

The phases of the calcined powders were identified by X-ray diffraction (XRD, XRD-6100, Shimadzu, Kyoto, Japan). The XRD pattern of the calcined powder is shown in Figure S1. For the structure refinement of the sintered samples, XRD data (MPXRD, X’Pert Pro, PANalytical, Almelo, The Netherlands) were collected at room temperature in the scan range of 2*θ* = 10°~100° with a scan step of 0.026° and then analyzed by Rietveld refinement using the FullProf program. The SC and the AQ samples for XRD analysis were heat-treated at 800 °C for 15 min without the silver electrode and cooled in the same manner as the SC and AQ samples. The microstructures of the sintered samples were imaged by scanning electron microscopy (SNE-4500M, SEC, Suwon-si, Republic of Korea). The dielectric constants at room temperature and temperature-dependent dielectric constants were measured at frequencies of 1 kHz–1 MHz using an impedance analyzer (4294A, Agilent, Santa Clara, CA, USA). The temperature-dependent dielectric constants were measured by increasing the temperature from room temperature to 700 °C with a rate of 1 °C/min. The ferroelectric P-E hysteresis characteristics were measured at room temperature in silicon oil using a ferroelectric tester (RT66A, Radiant, El Segundo, CA, USA) and a high-voltage amplifier (609E-6-L-CE, Trek, Lockport, NY, USA). The samples were poled at 120 °C in a silicon oil bath by applying a DC electric field of 3 kV/mm for 30 min. The electromechanical coupling factors (*k*_P_) were measured using an impedance analyzer with the resonance method.

## 3. Results and Discussion

**Figure 1:**
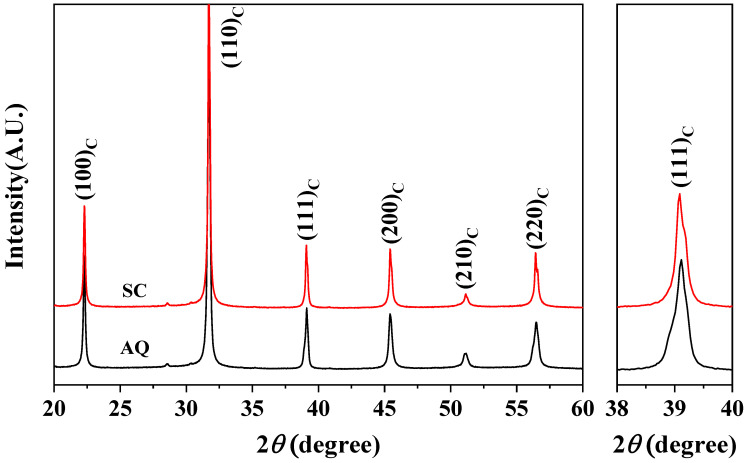
X-ray diffraction patterns of the SC and AQ 0.7BF-0.3BT ceramics.

Figure 1 shows XRD patterns of the (a) SC and (b) AQ 0.7BF-0.3BT ceramics, which were indexed based on a cubic structure. The detailed XRD patterns for the 2*θ* = 38–40° range are included in Figure 1 to confirm the splitting of the (111)_C_ diffraction peak due to rhombohedral distortion. Both samples show XRD patterns for a perovskite BF-BT solid solution with tiny impurity peaks around 2*θ* = 28.5° and 30.4°. The (111)_C_ diffraction peak of the AQ 0.7BF-0.3BT sample appears as the overlapped shape of two more diffraction peaks, while that of the SC sample looks like a single diffraction peak. It has been reported that a ferroelectric rhombohedral phase and a relaxor-like pseudo-cubic phase coexist in BF-BT ceramics with the MPB composition, and quenched samples show larger rhombohedral distortion or a larger portion of ferroelectric rhombohedral phase than the slowly cooled samples [16,17,25]. Although the XRD patterns in Figure 1 seem to be consistent with these previous reports, a precise structure refinement is required in order to accurately identify the crystal phases in BF-BT ceramics.

**Figure 2:**
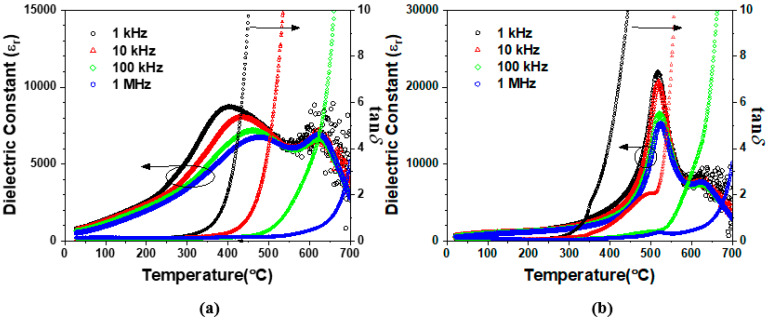
The change in dielectric constant with temperature in the (**a**) SC and (**b**) AQ 0.7BF-0.3BT ceramics.

Figure 2 shows the temperature dependence of the dielectric constants and loss tangents in the SC samples (a) and in the AQ samples (b). The loss tangent was increased abruptly above 300 °C due to the increase of the electric conductivity [16]. Two dielectric anomalies are observed in the SC samples: the broad frequency-dependent dielectric peak at low temperatures of around 300~500 °C (LT dielectric peak) and the frequency-independent dielectric peak at high temperatures of around 620 °C (HT dielectric peak). These two kinds of dielectric peaks have previously been observed in BF-BT ceramics around the MPB composition due to the coexistence of rhombohedral and pseudo-cubic phases [16]. These dielectric peaks were attributed to the phase transition of a pseudo-cubic phase with a relaxor behavior at low temperature and the phase transition of a ferroelectric rhombohedral phase at high temperature [16]. The peaks in Figure 2a coincide with this previous report, suggesting that a rhombohedral ferroelectric phase and a pseudo-cubic relaxor phase coexist in the SC 0.7BF-0.3BT ceramic. Figure 2b shows the temperature dependence of the dielectric constant in the AQ 0.7BF-0.3BT ceramic. Two types of dielectric anomalies are also shown in the AQ 0.7BF-0.3BT ceramic. However, unlike the SC sample, the LT dielectric peak at about 520 °C does not show a frequency dispersion. This result implies that the AQ sample consists of a pseudo-cubic phase and a rhombohedral phase. However, the pseudo-cubic phase, which shows an LT dielectric anomaly without frequency dispersion, is not a relaxor but a ferroelectric phase.

**Figure 3:**
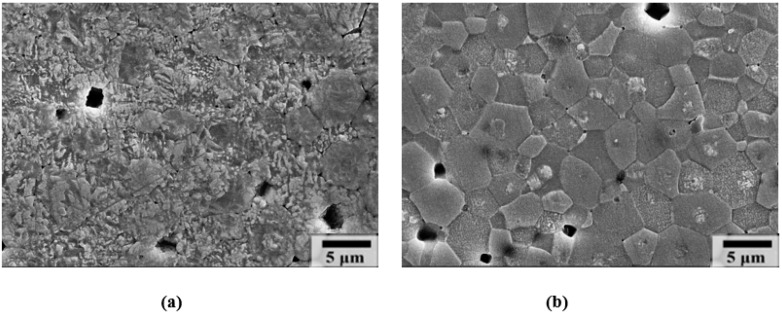
Chemically etched surfaces show ferroelectric domain patterns in (**a**) AQ and (**b**) SC 0.7BF-0.3BT ceramics.

Figure 3 shows the chemically etched surfaces of the polished (a) AQ and (b) SC 0.7BF-0.3BT ceramics. Ferroelectric domain patterns are clearly shown across the entire area of the AQ sample. In the SC sample, however, many grains show the domain patterns in the central area and featureless smooth surfaces in the edge areas. This microstructure suggests that a relaxor pseudo-cubic phase coexists with a ferroelectric rhombohedral phase in the SC sample. The core–shell structure with a BF-rich core and a BF-deficient shell has been observed in slowly cooled BF-BT ceramics [16,17,18,33,34]. It has been claimed that micro-diffusion during the slow cooling resulted in a BF-rich ferroelectric core and a BF-deficient relaxor shell [16]. However, chemical heterogeneity in the SC sample is still controversial, and the origin of the core–shell structure has not been clearly revealed [34,37].

The crystal structures of the SC and AQ 0.7BF-0.3BT ceramics were analyzed by Rietveld refinement using XRD data. The ferroelectric phase at high temperature is well known as a rhombohedral [11,12,13,14,15,16,17,18,19]. In this study, the pseudo-cubic phase with the LT dielectric anomaly is assigned to a non-rhombohedral phase, such as a cubic, tetragonal, or orthorhombic one. The XRD patterns were refined based on various two-phase models consisting of rhombohedral (R3c or R3m)–cubic (Pm-3m), rhombohedral (R3c or R3m)–tetragonal (P4mm), and rhombohedral (R3c or R3m)–orthorhombic (Pbnm or Pmc2_1_), as well as single-phase models. Overall, the R factors for two-phase models were lower than for those of single-phase models, as shown in Table 1 and Table 2, and Figures S2 and S3. The meanings of R factors are listed in Table S3. The summary of the structural refinements for the SC 0.7BF-0.3BT sample are shown in Table 1 and the Rietveld refinement profiles are displayed in Figure S2. The two-phase models consisting of rhombohedral (R3m or R3c) and orthorhombic phase (Pmc2_1_ or Pbnm) produced lower R factors than the other two-phase models, including rhombohedral–cubic (Pm-3m) and rhombohedral–tetragonal (P4mm). Among the rhombohedral–orthorhombic models, the super-structural rhombohedral phase (R3c, c~13.7Å) gave rise to slightly higher R-vales than the rhombohedral cell with a halved volume (R3m, c~6.87Å). The R3m–orthorhombic models show similar R-values when using both the non-polar orthorhombic (Pbnm) and the polar orthorhombic (Pmc2_1_) phase. The two-phase model consisting of the halved-rhombohedral cell (R3m) and the non-polar orthorhombic cell (Pbnm) was selected as the most suitable model for the SC 0.7BF-0.3BT sample in this work because of the strong frequency dependence of the LT dielectric peaks, as shown in Figure 2a. These structure analysis results for the SC 0.7BF-0.3BT sample suggest that the HT frequency-independent dielectric peak in Figure 2a resulted from the phase transition of the ferroelectric R3m phase to a paraelectric cubic structure. Additionally, the relaxor phase transition of the orthorhombic Pbnm phase is suggested to lead to the frequency-dependent broad LT dielectric peaks.

In this study, we first refined the pseudo-cubic phase in BF-BT ceramics as an orthorhombic Pbnm structure. This result is quite different from previous reports, in which the pseudo-cubic phase at the MPB has been reported to have a cubic or tetragonal structure [6,7,9,12,13,14,15,16,17,18,19,20,21,32]. However, the orthorhombic phase has frequently been reported in BiFeO_3_-based systems [38,39,40,41,42,43]. The crystal structure of BiFeO_3_ has been reported to change from a rhombohedral R3c to an orthorhombic Pbnm (or equivalently Pnma, SG # 62) when either the temperature increases above 825 °C or the hydrostatic pressure increases to approximately 10 GPa at room temperature [38]. We also note that the orthorhombic Pbnm phase has been observed in rare earth ions-doped BiFeO_3_, Bi_1-*x*_Ln*_x_*FeO_3_ (Ln = La, Nd, Sm, …) [40,41,42,43].

The summary of Rietveld refinements and the refinement profiles for the AQ 0.7BF-0.3BT sample are shown in Table 2 and Figure S3. The AQ sample shows lower R factors for the two-phase model consisting of the rhombohedral R3m and the orthorhombic phase (Pbnm or Pmc2_1_) than the other two-phase models. The rhombohedral–orthorhombic two-phase model shows slightly lower R factors when using the non-polar orthorhombic (Pbnm) phase than the polar orthorhombic (Pmc2_1_) phase. However, the LT dielectric characteristics with no frequency dispersion in Figure 2b suggest that the pseudo-cubic phase should be refined as a ferroelectric orthorhombic phase Pmc2_1_. Based on these results, we claim that a rhombohedral R3m and an orthorhombic Pmc2_1_ phases coexist in the AQ 0.7BF-0.3BT sample, and led to the two distinct frequency-independent dielectric anomalies observed in Figure 2b.

**Figure 4:**
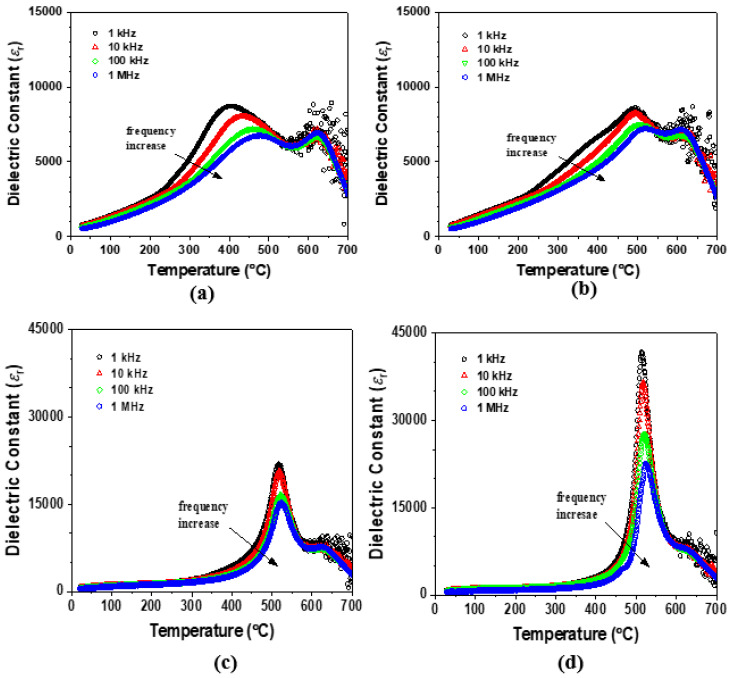
Temperature-dependent dielectric constants and loss tangents in (**a**) the SC sample before poling, (**b**) the SC sample after poling, (**c**) the AQ sample before poling and (**d**) the AQ sample after poling.

Figure 4 shows the change in the temperature-dependent dielectric constant after poling by applying a DC electric field of 3 kV/mm in the SC and AQ 0.7BF-0.3BT ceramics. As shown in Figure 4a,b, the frequency-dependent LT dielectric peak in the SC sample was partially changed after poling: the dielectric peak became sharper and had less frequency dependence in the temperature range of 400–500 °C, and the frequency dispersion of the dielectric peak remained in the temperature range of 200–400 °C. This result suggests that a part of the non-polar orthorhombic Pbnm phase changed to a polar Pmc2_1_ phase when the DC electric field was applied. On the other hand, the HT dielectric peak was not significantly changed by poling. In Figure 4c,d, we illustrate the change in the temperature-dependent dielectric constant after poling in the AQ 0.7BF-0.3BT ceramic. Notably, we observe an increase in the intensity of the LT dielectric peak after poling the AQ sample. The increase in the LT dielectric peak height seems to result from the rearrangement of ferroelectric domains and/or the change in the relative amounts of the rhombohedral and orthorhombic phases due to poling.

**Figure 5:**
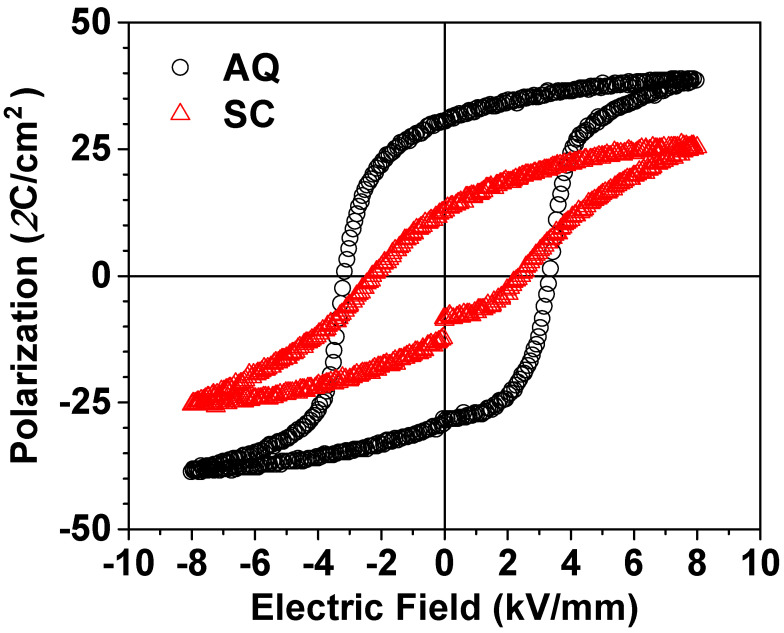
The ferroelectric P-E hysteresis curves of the SC and AQ 0.7BF-0.3BT ceramics.

Figure 5 shows the difference in the P-E hysteresis curve between the SC and AQ 0.7BF-0.3BT ceramics. The SC sample shows a slanted P-E curve and smaller polarization, whereas a well-saturated P-E hysteresis curve is observed in the AQ sample. The poor ferroelectric P-E hysteresis curve of the SC sample seems to be due to the non-polar Pbnm orthorhombic phase hindering the switching of the polarization from the applied electric field in the ferroelectric rhombohedral phase.

The piezoelectric properties of the AQ and SC 0.7BF-0.3BT ceramics are listed in Table 3. The AQ 0.7BF-0.3BT ceramic had a slightly larger dielectric constant (*ε*_r_) and a lower loss tangent (tan*δ*) than the SC sample. Table 3 illustrates that the AQ 0.7BF-0.3BT ceramic had a significantly higher piezoelectric constant (*d*_33_) and electromechanical coupling factor (*k*_P_) than the SC sample. The low *θ*_max_ of the SC sample suggests that the poor piezoelectric properties stem from the low degree of poling. The maximum phase (*θ*_max_) after poling has been reported to be a measure of the degree of poling [44]. The phase (*θ*) of a piezoelectric material changes from −90° in an unpoled state to 90° in an ideally poled state. The low degree of poling in the SC sample indicates that the dipole alignment along the applied electric field was hindered by the presence of the non-polar Pbnm orthorhombic phase. The AQ sample shows excellent piezoelectric properties: a *ε*_r_ of 780, a *d*_33_ of 191 pC/N and a *k*_P_ of 0.365.

**Figure 6:**
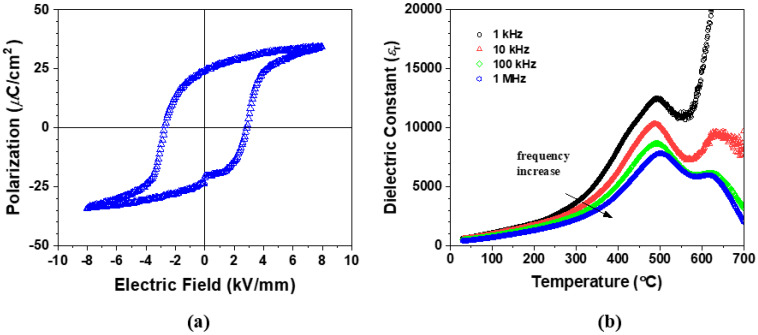
The P-E hysteresis curve (**a**) and the temperature-dependent dielectric constant (**b**) in the N2-SC sample.

Various mechanisms have been proposed for the origin of the quenching effect in the BF-BT ceramics. Many papers have cited the chemical heterogeneity or impurity phases from micro-diffusion during slow cooling as the origin of the quenching effect [6,16,32,33,34]. A strain gradient-induced polarization or flexoelectric effect has been reported to significantly influence the dielectric and ferroelectric properties in ferroelectric thin films and nano-particles [45,46,47,48]. The AQ sample may have the strain gradient from the surface to interior or the strain gradient near the phase boundaries between the rhombohedral and orthorhombic phases. However, the strain gradient-induced polarization has been reported to be very small even under high strain gradient in Pb(Zr,Ti)O_3_ ceramics [48,49]. The quenching effect in the AQ sample is not expected to be caused by the flexoelectric effect. The structure analysis in this work suggests that the enhanced properties of the quenched sample are closely related to the crystal structure of the pseudo-cubic phase. As a result, we attempted to examine the influence of the oxygen vacancy on the quenching effect. The BF-BT ceramic was annealed at 800 °C for 1 h in N_2_ atmosphere and then slowly cooled at a rate of 2 °C/minute to room temperature in N_2_ atmosphere (N2-SC sample). The crystal structure of the N2-SC sample was analyzed by XRD. Any additional phase and a phase decomposition were not observed in the XRD pattern (Figure S3). The N2-SC sample also shows lower R factors for the two-phase model consisting of the rhombohedral (R3m or R3c) and the orthorhombic phases (Pmc2_1_ or Pbnm) than the other two-phase models, including rhombohedral–cubic (Pm-3m) and rhombohedral–tetragonal (P4mm) (Table S4). The R3c rhombohedral phase shows slightly lower R-vales than the R3m rhombohedral cell in the rhombohedral–orthorhombic models, and the R3m–orthorhombic models display similar R-values when using the non-polar orthorhombic (Pbnm) compared to the polar orthorhombic (Pmc2_1_) phase. Figure 6a shows that the P-E hysteresis curve of the N2-SC sample is well-saturated and more closely resembles that of the AQ sample than the SC sample. Figure 6b displays that the change in the dielectric constant with temperature in the N2-SC sample has a similar shape to that of the AQ sample. As a result, we conclude that the concentration of the oxygen vacancies had a more direct impact on the enhanced piezoelectric properties of the AQ sample than the cooling rate.

It has been reported that bismuth and oxygen vacancies are generated due to the evaporation of bismuth oxide during the sintering process in BF-BT ceramics [50]. The oxygen vacancies are partially filled by oxygen diffusion in an oxygen-rich atmosphere through the following equations [50]:

(1) $$2{Bi}_{Bi}+3O_{O}\;\to 2V_{Bi}^{\prime \prime \prime }+3V_{O}^{{}^{\circ}{}^{\circ}}+{Bi}_{2}O_{3}(g)$$

(2) $$V_{O}^{{}^{\circ}{}^{\circ}}+\frac{1}{2}O_{2}\left(g\right)\to O_{O}+2h^{{}^{\circ}}\;$$

In accordance with Equation (2), the BF-BT ceramic has been reported to show p-type conduction in air [45]. During the slow cooling in air, oxygen gas diffuses into the sample, fills the oxygen vacancies, and generates electron holes. This is consistent with the report that the SC sample has larger leakage current than the quenched sample [6]. On the other hand, a large concentration of oxygen vacancies is expected to remain at room temperature in the AQ and N2-SC samples. The large concentration of oxygen vacancies seems to lead to a non-centrosymmetric polar Pmc2_1_ structure, which is more distorted than a symmetric Pbnm structure in the SC sample. A further study is required to reveal the detailed mechanism by which the oxygen vacancies lead to the structure change.

## 4. Conclusions

The crystal structures and the dielectric behavior around phase transition temperatures were investigated in the quenched (AQ) and slowly cooled (SC) 0.7BF-0.3BT ceramics. By using Rietveld structure refinement, we determine that a rhombohedral and an orthorhombic phase coexist in 0.7BF-0.3BT ceramics. The crystal structure of the SC samples was refined as a rhombohedral R3m and a non-polar orthorhombic Pbnm, whereas the AQ sample’s crystal structure was refined as a rhombohedral R3m and a polar orthorhombic Pmc2_1_. The SC sample showed two dielectric anomalies: a frequency-independent dielectric peak at 620 °C due to the ferroelectric–paraelectric phase transition of the rhombohedral phase and a broad dielectric peak with strong frequency dispersion around 200~500 °C due to the relaxor phase transition of a non-polar orthorhombic phase. By contrast, two sharp frequency-independent dielectric peaks were observed at about 620 °C and 520 °C in the AQ sample, which correspond to the Curie temperatures of a rhombohedral and a polar orthorhombic structure, respectively. The AQ sample showed better ferroelectric and piezoelectric properties than the SC sample because the dipole alignment from the applied electric field was hindered by a non-polar orthorhombic phase in the SC sample. The 0.7BF-0.3BT ceramic that was slowly cooled in nitrogen atmosphere showed a similar P-E hysteresis curve and temperature-dependent dielectric constant to those seen in the AQ sample. This result suggests that a large concentration of oxygen vacancies in the AQ sample leads to a more distorted polar orthorhombic phase and better piezoelectric properties.

## Figures and Tables

**Table 1:** The summary of the structural refinements for the SC 0.7BF-0.3BT ceramic.

Phase	Fraction	Lattice Parameters			R Factors				
(SG)		a (Å)	b (Å)	c (Å)	R_p_	R_wp_	R_exp_	R_b_	R_f_
R3c	0.41	5.6111 (15)	5.6111 (15)	13.7452 (53)	8.53	11.22	2.54	5.81	4.95
Pm$\bar{3}$m	0.59	3.9624 (5)	3.9624 (5)	3.9624 (5)				5.85	4.12
R3m	0.36	5.6027 (27)	5.6027 (27)	6.8579 (20)	7.55	9.64	2.54	5.90	4.93
Pm$\bar{3}$m	0.64	3.9605 (4)	3.9605 (4)	3.9605 (4)				5.38	3.74
R3c	0.37	5.6006 (18)	5.6006 (18)	13.7248 (98)	7.51	9.77	2.54	6.98	6.35
P4mm	0.63	3.9603 (3)	3.9603 (3)	3.9603 (3)				6.91	5.52
R3m	0.34	5.6222 (10)	5.6222 (10)	6.8569 (11)	8.16	10.66	2.54	5.60	4.94
P4mm	0.66	3.9576 (6)	3.9576 (6)	3.9617 (5)				5.26	4.10
R3c	0.20	5.6020 (11)	5.6020 (11)	13.7495 (51)	5.77	7.45	2.54	5.17	3.35
Pmc21	0.80	7.9193 (5)	5.5981 (4)	5.6049 (6)				5.00	3.58
R3m	0.35	5.6009 (7)	5.6009 (7)	6.8717 (30)	5.51	7.25	2.53	4.43	3.51
Pmc21	0.65	7.9190 (5)	5.5968 (4)	5.6029 (14)				4.25	3.75
R3c	0.23	5.6024 (9)	5.6024 (9)	13.7442 (77)	5.78	7.44	2.54	5.15	3.63
Pbnm	0.77	5.5983 (4)	7.9202 (5)	5.6051 (10)				5.13	3.57
R3m	0.41	5.6018 (5)	5.6018 (5)	6.8743 (23)	5.47	7.23	2.54	3.77	3.38
Pbnm	0.59	5.5988 (4)	7.9210 (5)	5.6028 (14)				3.87	3.52

**Table 2:** The summary of the structural refinements for the AQ 0.7BF-0.3BT ceramic.

Phase	Fraction	Lattice Parameters			R Factors				
(SG)		a (Å)	b (Å)	c (Å)	R_p_	R_wp_	R_exp_	R_b_	R_f_
R3c	0.42	5.5978 (20)	5.5978 (20)	13.7985 (35)	5.47	7.09	2.40	3.60	2.16
Pm3m	0.58	3.9630 (4)	3.9630 (4)	3.9630 (4)				2.81	1.96
R3m	0.37	5.6029 (19)	5.6029 (19)	6.8879 (15)	5.56	7.24	2.41	2.85	2.34
Pm$\bar{3}$m	0.63	3.9599 (3)	3.9599 (3)	3.9599 (3)				2.79	1.91
R3c	0.31	5.5966 (27)	5.5966 (27)	13.7839 (35)	5.47	7.09	2.40	2.58	2.16
P4mm	0.69	3.9597 (6)	3.9597 (6)	3.9641 (12)				2.81	1.96
R3m	0.35	5.6012 (15)	5.6012 (15)	6.8887 (13)	5.46	7.27	2.40	3.01	2.91
P4mm	0.65	3.9597 (19)	3.9597 (19)	3.9596 (40)				3.06	2.63
R3c	0.21	5.6104 (11)	5.6104 (11)	13.7321 (122)	4.55	6.05	2.40	2.99	2.38
Pmc21	0.79	7.9190 (7)	5.5915 (5)	5.6150 (15)				3.01	2.28
R3m	0.29	5.6079 (9)	5.6079 (9)	6.8795 (35)	4.54	5.99	2.40	2.55	2.20
Pmc21	0.71	7.9205 (8)	5.5908 (6)	5.6105 (15)				2.62	2.17
R3c	0.20	5.6109 (12)	5.6109 (12)	13.7635 (42)	4.41	5.91	2.40	2.44	2.37
Pbnm	0.80	5.5920 (6)	7.9206 (7)	5.61008 (9)				2.37	2.28
R3m	0.21	5.6089 (12)	5.6089 (12)	6.8811 (21)	4.29	5.83	2.40	2.61	2.01
Pbnm	0.79	5.5921 (5)	7.9206 (7)	5.6116 (8)				2.41	1.90

**Table 3:** Piezoelectric properties of the AQ and SC 0.7BF-0.3BT ceramics at room temperature. The *ε*_r_ and tan δ were measured at 1 kHz.

Sample	*ε*_r_	tan δ	*d*_33_ (pC/N)	*k*_p_	*θ*_max_ (°)
AQ	780	0.053	191	0.365	57.2
SC	759	0.057	65	0.204	−43.9

## Data Availability

The original contributions presented in the study are included in the article/supplementary material, further inquiries can be directed to the corresponding author.

## Supplementary Materials

The following supporting information can be downloaded at: https://www.mdpi.com/article/10.3390/ma17184492/s1.
