# Supplementary material for: Crystal Structures and Piezoelectric Properties of Quenched and Slowly-Cooled BiFeO3-BaTiO3 Ceramics
Source: Materials (Basel). 2024 Sep 13;17(18):4492. doi: 10.3390/ma17184492 (PMC11432820; doi:10.3390/ma17184492)
Supplement: Supplementary file 1 [file materials-17-04492-s001.zip › materials-3182089-supplementary.pdf]

Supplementary Materials

Crystal Structures and Piezoelectric Properties of Quenched and Slowly-Cooled $BiFeO_3$-$BaTiO_3$ Ceramics

Su Hwan Go[1], Kangsan Kim[1], Ye Rok Choi[2], Jeong-Seog Kim[1], and Chae Il Cheon[1,2,*]

[1]Department of Materials Science & Engineering, Hoseo University, Asan 31499, Korea

[2]Department of Electronic Materials Engineering, Hoseo University, Asan 31499, Korea

*Corresponding author : cicheon@hoseo.edu

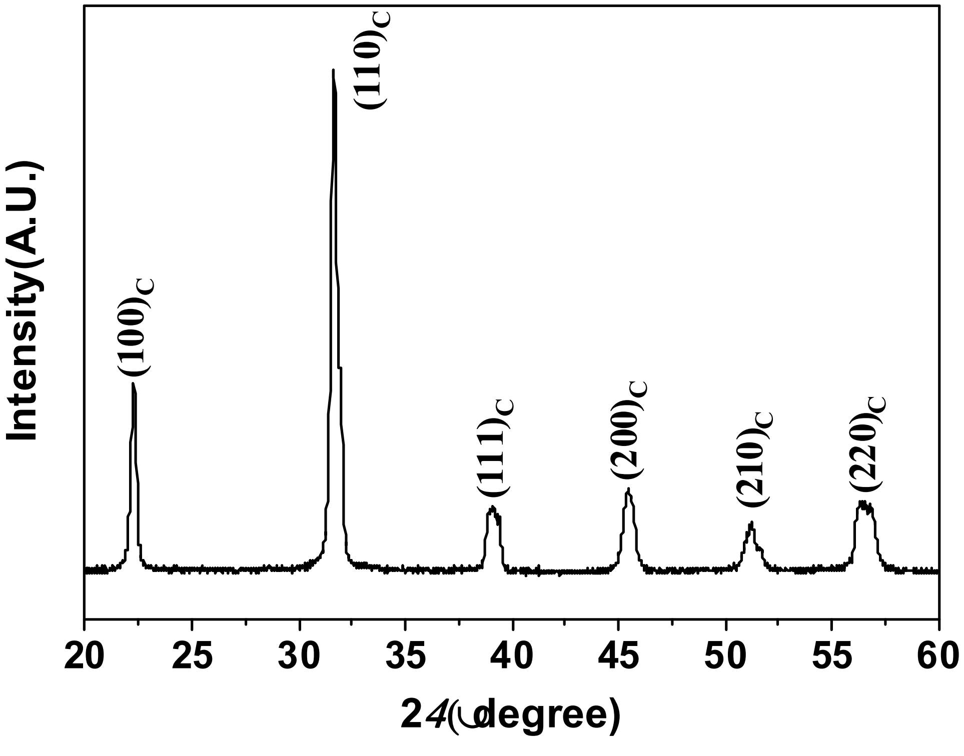

Fig. S1 XRD pattern of the calcined powder.

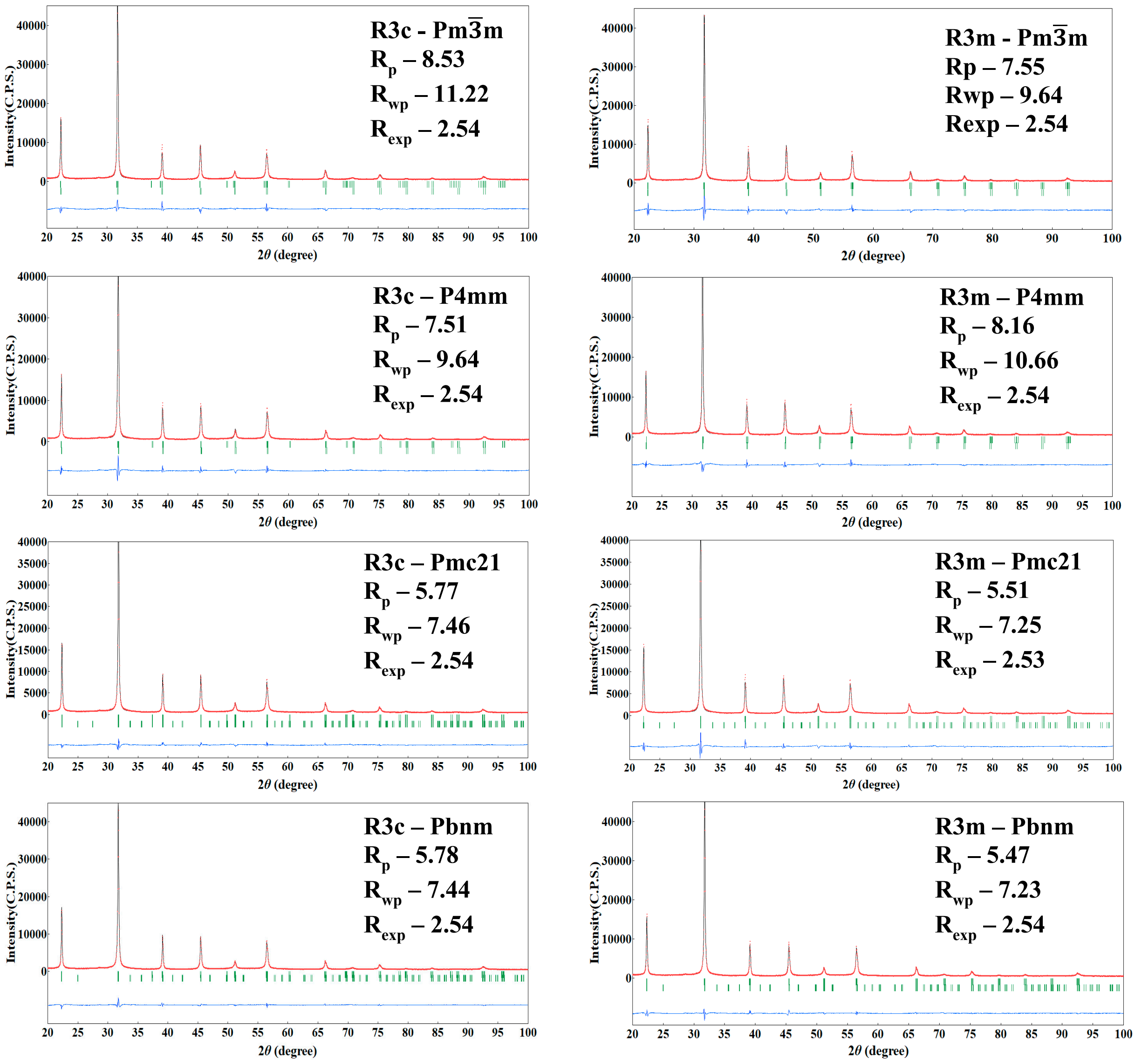

Fig. S2 Rietveld refinement profiles for two-phase model in the SC sample.

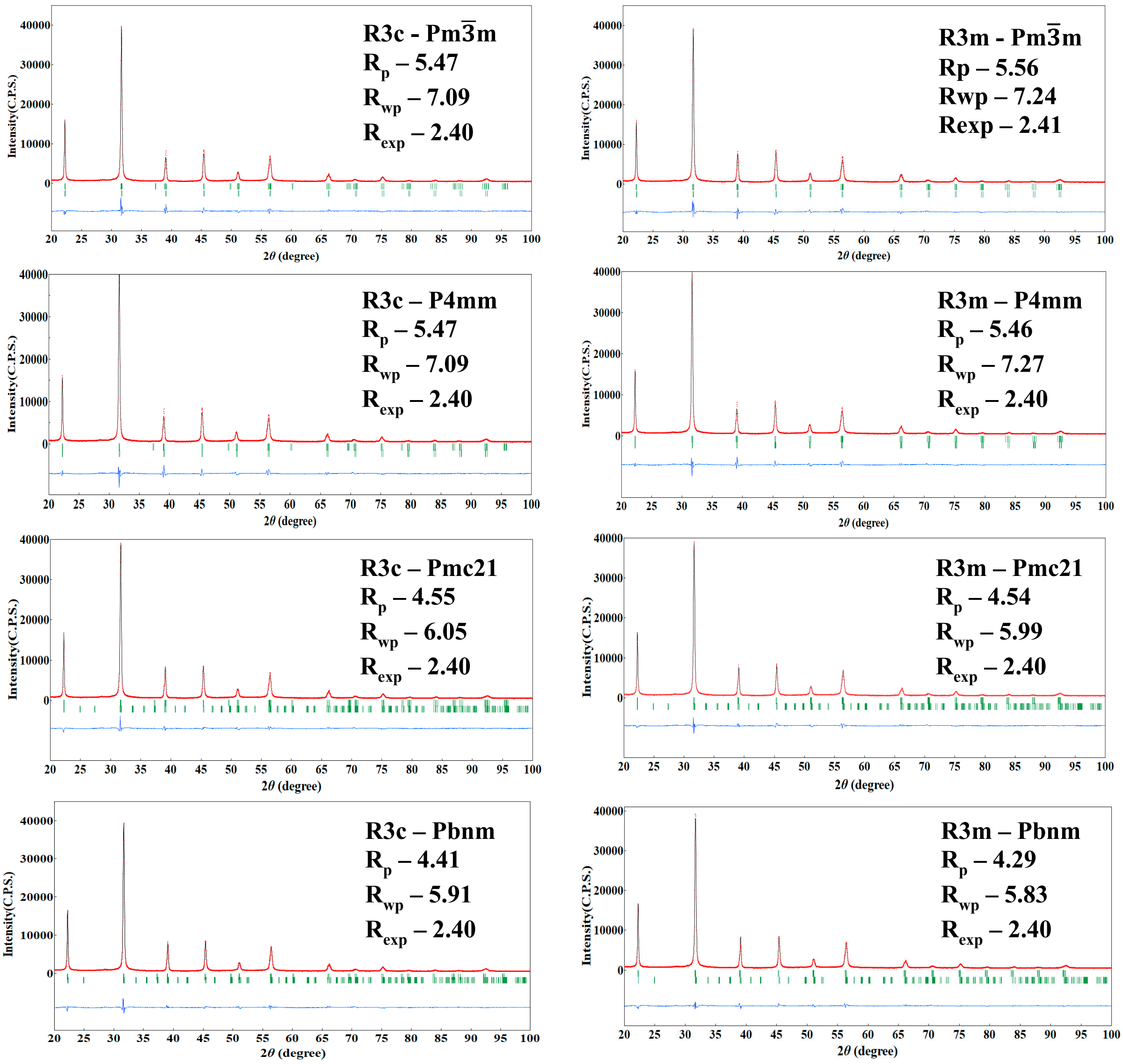

Fig. S3 Rietveld refinement profiles for two-phase model in the AQ sample.

Table S1. The summary of the structural refinements for a single-phase model in the SC 0.7BF-0.3BT ceramic .

**phase**	**lattice parameters**			**R factors**				
**(SG)**	**a (Å)**	**b (Å)**	**c (Å)**	R_p	R_{wp}	R_{exp}	R_b	R_f
R3c	5.5972(5)	5.5972(5)	13.7147(19)	7.23	9.32	2.54	5.77	4.68
R3m	5.5980(10)	5.5980(10)	6.8663(11)	7.84	10.19	2.54	6.95	4.31
Pmc21	7.9167(9)	5.5975(7)	5.6039(7)	6.72	8.57	2.54	5.11	3.79
Pbnm	5.6002(4)	7.9203(5)	5.6067(5)	7.24	9.12	2.53	5.96	5.11
P4mm	3.9603(4)	3.9603(4)	3.9639(5)	8.08	10.39	2.54	6.84	5.89
$Pm\bar{3}m$	3.9575(7)	3.9575(7)	3.9575(7)	10.56	13.93	2.54	10.05	7.13

Table S2. The summary of the structural refinements for a single-phase model in the AQ 0.7BF-0.3BT ceramic.

**phase**	**lattice parameters**			**R factors**				
**(SG)**	**a (Å)**	**b (Å)**	**c (Å)**	R_p	R_{wp}	R_{exp}	R_b	R_f
R3c	5.5947(10)	5.5947(10)	13.7583(19)	6.42	8.31	2.41	4.97	4.03
R3m	5.6073(6)	5.6073(6)	6.8504(8)	5.52	7.31	2.40	3.50	3.46
Pmc21	7.9178(9)	5.5921(6)	5.6166(6)	5.25	6.86	2.40	3.62	2.73
Pbnm	5.5925(5)	7.9191(8)	5.6163(5)	5.25	7.73	2.40	4.06	3.24
P4mm	3.9590(2)	3.9590(4)	3.9673(5)	5.84	7.73	2.40	4.06	3.24
$Pm\bar{3}m$	3.9618(2)	3.9618(2)	3.9618(2)	6.81	9.03	2.41	6.47	3.67

Table S3 The meaning of R factors in the structural refinement results.

$\boldsymbol{R_p}$(**Profile R − factor**): The R−factor representing the absolute difference between the experimental and model data. $[\frac{\sum|Y_{oi}-Y_{ci}|}{\sum|Y_{oi}|}]$

$\boldsymbol{R_{wp}}$(**Weighted pattern**): The R−factor that calculates the weighted difference between the experimental and model data. $[\left[\frac{\sum\omega_i(|Y_{oi}-Y_{ci}|)^2}{\sum\omega_i|Y_{oi}|^2}\right]^{1/2}]$

$\boldsymbol{R_{exp}}$(**Expexted R − factor**): the best value R for a data set. $[\left[\frac{(N-P+C)}{\sum\omega_i|Y_{oi}|}\right]^{1/2}]$

$\boldsymbol{R_b}$(**Bragg R factor**): The R−factor for a specific crystal phase. $[\frac{\sum|I_k-I_{kc}|}{\sum I_k}]$

$\boldsymbol{R_f}$(**Structure R factor**): The R−factor representing the difference between observed and calculated structure factors. $[\frac{\sum|F_o-F_c|}{\sum F_o}]$

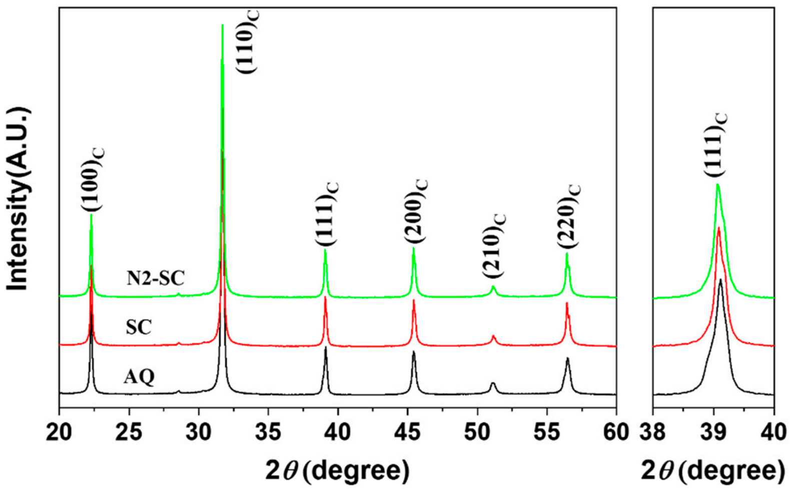

Fig. S4 X-ray diffraction patterns of the N2-SC, SC and AQ 0.7BF-0.3BT ceramics.

Table S4. The summary of the structural refinements for the N2-SC 0.7BF-0.3BT ceramic.

phase (SG)	fraction	lattice parameters a (Å)	b (Å)	c (Å)	R factors R_p	R_{wp}	R_{exp}	R_b	R_f
R3c	0.28	5.6009(29)	5.6009(29)	13.8192(41)	8.30	10.62	2.51	8.31	7.92
$Pm\bar{3}m$	0.72	3.9627(3)	3.9627(3)	3.9627(3)				7.26	5.98
R3m	0.39	5.6032(31)	5.6032(31)	6.8590(23)	7.38	9.82	2.52	5.62	4.52
$Pm\bar{3}m$	0.61	3.9607(6)	3.9607(6)	3.9607(6)				5.24	3.52
R3c	0.30	5.6064(25)	5.6064(25)	6.8712(30)	8.77	11.10	2.52	8.53	9.37
P4mm	0.70	3.9612(-)	3.9612(-)	3.9612(-)				7.72	7.52
R3m	0.29	5.6050(30)	5.6050(30)	6.8722(25)	7.69	9.81	2.52	5.00	4.87
P4mm	0.71	3.9613(13)	3.9613(13)	3.9605(26)				4.41	3.83
R3c	0.28	5.6024(8)	5.6024(8)	13.7234(137)	5.33	7.01	2.51	4.51	3.58
Pmc21	0.72	7.9210(5)	5.5988(3)	5.6080(22)				4.47	3.50
R3m	0.33	5.6011(6)	5.6011(6)	6.8712(30)	5.50	7.36	2.51	3.48	3.13
Pmc21	0.67	7.9197(5)	5.5960(4)	5.6041(15)				3.45	3.18
R3c	0.26	5.6041(7)	5.6041(7)	13.7596(677)	5.45	7.11	2.51	4.34	3.14
Pbnm	0.75	5.5993(3)	7.9222(4)	5.6050(5)				4.25	3.22
R3m	0.29	5.6042(7)	5.6042(7)	6.8819(24)	5.37	7.14	2.51	3.21	3.71
Pbnm	0.71	5.5993(4)	7.9224(5)	5.6039(10)				3.11	3.34
